# Evaluation of Constitutive Models for Low-Temperature Performance of High-Modulus Modified Asphalt: A BBR Test-Based Study

**DOI:** 10.3390/ma18173963

**Published:** 2025-08-24

**Authors:** Chao Pu, Bingbing Lei, Zhiwei Yang, Peng Yin

**Affiliations:** 1 School of Infrastructure Engineering, Dalian University of Technology, Dalian 116024, China; puchao19950906@163.com; 2 Xinjiang Key Laboratory for Safety and Health of Transportation Infrastructure in Alpine and High-Altitude Mountainous Areas, Urumqi 830000, China; 3 Xinjiang Transport Planning Survey and Design Institute Co., Ltd., Urumqi 830000, China; 241081401006@lut.edu.cn; 4 School of Civil Engineering, Lanzhou University of Technology, Lanzhou 730050, China; 5 Key Laboratory of Ecological Safety and Sustainable Development in Arid Lands, Xinjiang Institute of Ecology and Geography, Chinese Academy of Sciences, Urumqi 830011, China; 6 University of Chinese Academy of Sciences, Beijing 100049, China

**Keywords:** high-modulus asphalt, BBR test, constitutive model, creep behavior, GRA model

## Abstract

High-modulus asphalt, with its excellent fatigue resistance and high-temperature resistance, is gradually becoming a preferred material for the development of durable asphalt pavements. However, its poor low-temperature performance has become one of the key bottlenecks restricting its wide application. In recent years, in-depth analysis of the mechanism underlying the changes in the low-temperature performance of high-modulus asphalt has gradually become a research focus in the field of asphalt pavements. Accordingly, this study selected four representative high-modulus asphalts, conducted bending beam rheometer (BBR) tests to obtain their low-temperature creep parameters, and used three viscoelastic constitutive models to investigate their low-temperature constitutive relationships. Grey relational analysis (GRA) was further applied to evaluate the models. The results show that, when evaluating the low-temperature performance of high-modulus asphalt, the elastic and viscous parameters variation laws, for the three-parameter solid (TPS) model and four-parameter solid (FPS) model, are not obvious and have large fluctuations, and the accuracy of the fitting curves is relatively low, while the Burgers model has extremely high fitting accuracy, with small parameter fluctuations and significant regularity. The GRA model reveals that the Burgers model is more suitable than the TPS and FPS models for describing the low-temperature creep behavior of high-modulus asphalt, which further confirms the reliability of using the Burgers model to evaluate the low-temperature performance of high-modulus asphalt.

## 1. Introduction

In the modern urban transportation system, asphalt pavement is an essential infrastructure for people’s travel, bearing heavy traffic pressure and enduring continuously increasing vehicle loads [1,2,3]. However, for a long time, asphalt pavement has been subjected to erosion from harsh environmental conditions and ever-increasing traffic loads, which continuously elevates the risk of road damage and deterioration, accompanied by rising costs for road maintenance and repair [4,5,6]. Therefore, the research and development of durable asphalt pavements have become a key link in ensuring road quality and extending their service life [7,8]. In recent years, high-modulus asphalt has gradually emerged as an ideal choice for the development of durable asphalt pavements, due to its excellent high-temperature performance and fatigue resistance. It is worth mentioning that durable asphalt pavement refers to asphalt pavement that can maintain stable service performance and extend its service life under the action of long-term traffic loads and natural environments. The main types of polymers that are commonly used to modify asphalt to improve its durability include styrene-butadiene-styrene block copolymer (SBS), ethylene-vinyl acetate copolymer (EVA), etc., and these can significantly enhance the deformation resistance, fatigue resistance, and aging resistance of asphalt. However, relevant studies have indicated that high-modulus asphalt generally exhibits poor low-temperature performance, which has become a significant factor restricting its application as a material for durable asphalt pavements [9,10,11]. Against this backdrop, it is crucial to conduct in-depth research on the mechanism underlying the changes in the low-temperature performance of high-modulus asphalt. This not only provides guidance for improving the low-temperature performance of high-modulus asphalt to a certain extent but also contributes to promoting the sustainable development of durable asphalt pavements.

Currently, research on the low-temperature performance of asphalt mainly relies on the BBR test and physical properties tests such as penetration and softening point tests. However, the penetration and softening point tests can only reflect the ductility of asphalt in low-temperature environments within a certain range, and have significant limitations and are relatively one-sided in evaluating low-temperature performance [12,13]. In contrast, the BBR test characterizes the low-temperature performance of asphalt by measuring its creep behavior at low temperatures, which is more comprehensive and effective in evaluation, and it has thus become the primary method for evaluating the low-temperature performance of asphalt [14,15]. Liu [16] evaluated the variation in low-temperature performance of polyphosphoric acid-modified asphalt with the aging degree using the BBR test, and found that the incorporation of polyphosphoric acid modifier could effectively improve the low-temperature performance of asphalt and reduce the sensitivity of asphalt’s low-temperature performance to its aging degree. Mohammed [17] modified asphalt with two types of nanomaterials and characterized the low-temperature performance of the two modified asphalts using the BBR test. It was found that, although the incorporation of nanomaterials could effectively enhance the high-temperature performance and fatigue performance of asphalt, it would cause deterioration in the low-temperature performance of asphalt. Yan [18] investigated the recovery effect of waste cooking oil on the performance of aged asphalt, and used the BBR test to focus on observing the variation trend of the low-temperature performance of aged asphalt before and after regeneration. It was found that waste cooking oil improved the low-temperature cracking resistance of aged asphalt by reducing the creep stiffness and creep rate. Zhang [19] studied the modification effect of SBS modifier on different types of base asphalts and used the BBR test to investigate the low-temperature performance of two types of SBS modified asphalts. It was found that the low-temperature performance of both modified asphalts was significantly improved, but the improvement effect of SBS modifier on the low-temperature performance of asphalt weakened as the test temperature decreased. Notani [20] recovered carbon powder through functional modification of asphalt and evaluated the low-temperature performance of the modified asphalt via BBR tests and viscoelastic modeling methods. It was found that carbon powder could serve as a potential option to improve the low-temperature performance of asphalt. Liu [21] investigated the low-temperature performance of basalt fiber–rubber composite-modified asphalt using BBR tests and found that basalt fiber would deteriorate the low-temperature performance of asphalt to a certain extent. Xu [22] studied the prediction of asphalt’s low-temperature performance by combining BBR and DSR tests, and proposed a method for predicting the low-temperature performance of asphalt based on a rheological model. Marasteanu [23] conducted a comprehensive analysis of asphalt’s behavior at low temperatures and further discussed the development prospects of testing methods for asphalt’s low-temperature performance by combining BBR tests with various low-temperature models. The above research results indicate that the BBR test has become the primary research method for evaluating the low-temperature performance of asphalt at present. However, most current studies on low-temperature performance only evaluate asphalt’s low-temperature performance through low-temperature creep parameters, while in-depth analysis of the constitutive relationship of asphalt’s low-temperature performance is still lacking.

The low-temperature performance of asphalt materials is directly related to the crack resistance of pavements in cold environments [24]. An in-depth investigation into the constitutive relationship of their low-temperature performance can reveal the deformation laws and performance evolution mechanisms of asphalt under different low-temperature conditions from the perspective of mechanical response mechanisms, providing a theoretical basis for the targeted improvement of asphalt’s low-temperature crack resistance [25]. Especially for high-modulus asphalt, although it has become an important choice for long-life pavements due to its excellent high-temperature stability and durability, the issue of low-temperature brittleness has always been a key bottleneck restricting its popularization and application [26]. Therefore, conducting research on the constitutive relationship of the low-temperature performance of high-modulus asphalt holds significant practical significance. Against this backdrop, this study selects four representative high-modulus asphalts (a high-modulus agent-modified asphalt; a high-content SBS modifier-modified asphalt; a waste rubber powder composite-modified high-modulus asphalt; and a high-modulus asphalt prepared from hard asphalt) as research objects, determines their low-temperature creep characteristic parameters via BBR tests, and, with these as fundamental data, introduces three typical viscoelastic constitutive models, respectively, to systematically investigate their low-temperature viscoelastic constitutive behaviors. To screen out the model most suitable for characterizing the low-temperature creep properties of high-modulus asphalt, this study further employs the GRA model to quantitatively evaluate the three constitutive models based on dimensions such as parameter stability, fitting accuracy, and significance of physical meaning. This study aims to clarify the low-temperature constitutive relationship and applicable models of high-modulus asphalt, thereby providing a scientific reference for the optimization of the low-temperature performance, material selection, and engineering application of high-modulus asphalt and facilitating its wider application in durable pavement engineering.

## 2. Materials and Methods

### 2.1. Asphalt

**Figure 1:**
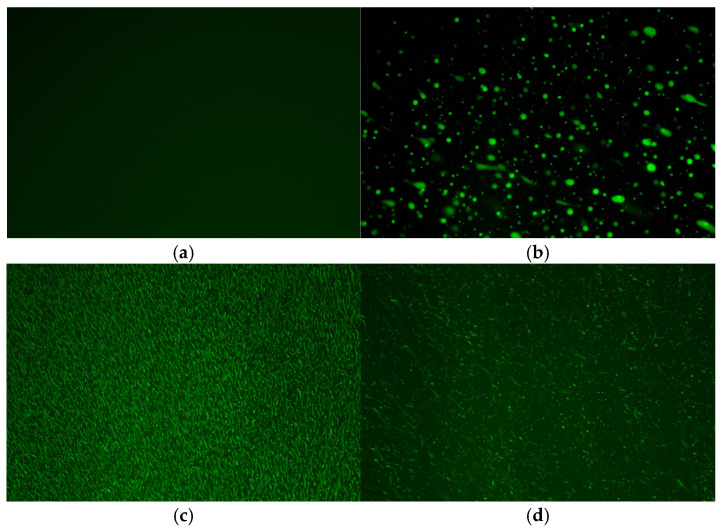
Microscopic images of several asphalts: (**a**) HGA; (**b**) WRA; (**c**) SBA; (**d**) HMA.

To investigate the constitutive relationship of the low-temperature performance of high-modulus asphalt, this study selected four representative asphalts for analysis, namely high-modulus agent-modified asphalt (HMA), high-content SBS modifier-modified asphalt (SBA), waste rubber powder composite-modified high-modulus asphalt (WRA), and high-modulus asphalt prepared from hard asphalt (HGA). Their basic properties are shown in Table 1, and microscopic images of them are shown in Figure 1.

### 2.2. BBR Test

The BBR test is an experimental method for evaluating the rheological properties of materials, and is mainly used to measure the deformation and stress response of materials under bending loading. In this study, the BBR test was employed to investigate the low-temperature creep behavior and mechanical response of several high-modulus asphalts, and the constitutive relationships of these high-modulus asphalts were established using time–history curves of creep behavior obtained during the tests [27,28]. In accordance with the specifications of JTG E20-2011, the BBR test was conducted on the high-modulus asphalts, with test temperatures set at −6 °C, −12 °C, and −18 °C. The low-temperature performance of the asphalts was evaluated based on the creep stiffness (S) and creep rate (m) derived from the tests.

## 3. Results

### 3.1. BBR Test Results

**Figure 2:**
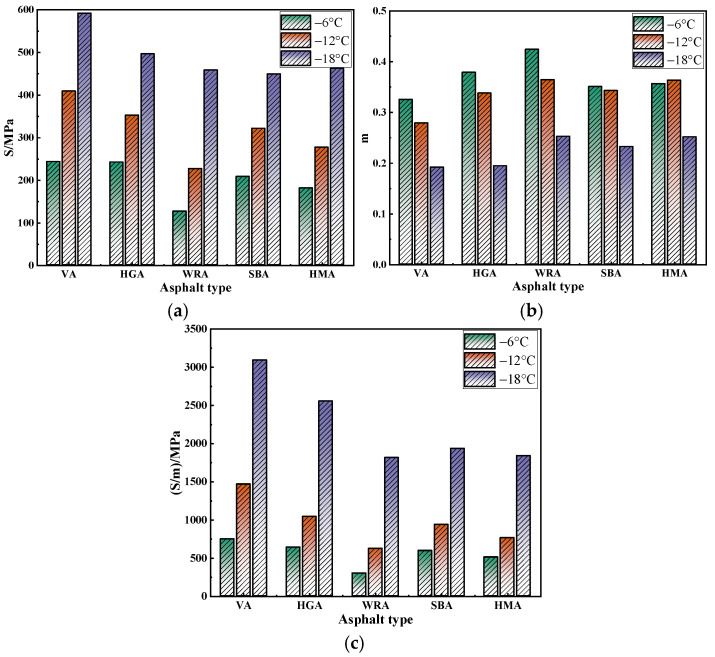
The results of BBR test: (**a**) S; (**b**) m; (**c**) S/m.

In this study, the low-temperature performance of several high-modulus asphalts was characterized via BBR tests and evaluated using S and m [29,30], with the results being shown in Figure 2.

As shown in Figure 2, the S values and m values of the several asphalts exhibit a similar variation trend: as the temperature decreases, the S values gradually increase while the m values gradually decrease. The lower the S value and the higher the m value, the poorer the low-temperature performance of the asphalt. This indicates that a decrease in temperature has an adverse effect on the low-temperature performance of high-modulus asphalt. In addition, it can be observed that the order of S values among the high-modulus asphalts is WRA < HMA < SBA < HGA, while the order of m values is WRA > HMA > SBA > HGA. This indicates that WRA exhibits the best low-temperature performance, whereas HGA shows the poorest. This is because WRA is produced via waste rubber powder composite modification. Waste rubber powder can fill the voids in asphalt, enhancing the interlocking and anchoring effects between asphalt and waste rubber powder. Moreover, waste rubber powder has high elastic recovery, which can effectively promote the elastic behavior of asphalt. Although the S values of HMA and SBA are slightly lower than those of WRA, the difference is not significant. This is because HMA and SBA are mainly prepared by compounding polymer modifiers. When polymer modifiers are miscible with asphalt, they can form a relatively stable three-dimensional network structure in asphalt, thereby enhancing its low-temperature deformation resistance. HGA exhibits the poorest low-temperature performance because it is a hard asphalt with no modified materials participating in reactions within it. Its high modulus mainly stems from the low proportion of light components in the asphalt. At low temperatures, the brittleness of the asphalt increases, and there are no modified materials in the asphalt to enhance its deformation resistance when it is subjected to external forces, which results in it having poorest low-temperature performance. In addition, it can be observed that there are contradictions in the test results of some asphalts, as there are differences in the order of changes between S values and m values. This indicates that there are certain limitations in evaluating low-temperature performance using only S values or m values.

Therefore, drawing on relevant research experience [31], this study uses the S/m value to evaluate low-temperature performance. As shown in Figure 2c, the variation trend of the S/m value is similar to that of the S value: as the temperature decreases, the S/m value of the several asphalts all gradually increase, which further confirms the adverse effect of temperature reduction on low-temperature performance. In addition, the order of the S/m values among the asphalts is WRA < HMA < SBA < HGA, which is consistent with the previous analysis results. According to the specification requirements of AASHTO M320 (m ≥ 0.3; S ≤ 300 MPa), it can be found that the low-temperature limit temperature of WRA and HMA is −12 °C, while that of SBA and HGA is −6 °C. However, compared with HGA, SBA is very close to the specification values. This indicates that, in practical applications, high-modulus asphalt prepared with waste rubber powder can achieve a relatively better low-temperature performance, followed by that prepared with polymer modifiers, while the low-temperature performance of hard asphalt is relatively poor.

### 3.2. Viscoelastic Constitutive Models of High-Modulus Asphalt

Asphalt is essentially a viscoelastic material. Compared with ordinary asphalt, high-modulus asphalt exhibits more significant high-temperature performance and fatigue performance due to its higher elastic modulus [32]. However, this also results in a lower proportion of viscoelastic components in it, which makes it tend to be more elastic [33,34]. Therefore, the mechanical behavior of high-modulus asphalt at low temperatures can be described by series-connected viscoelastic mechanical elements. For this purpose, this study uses three typical viscoelastic constitutive models to describe the low-temperature mechanical behavior of high-modulus asphalt, thereby elaborating on its constitutive relationship. These three typical viscoelastic constitutive models are the Burgers model, TPS model, and FPS model, respectively. These models are selected to construct the constitutive relationship of asphalt at low temperatures because they can accurately characterize the viscoelasticity (coupling of elasticity and viscosity) of asphalt at low temperatures, match the time-dependent characteristics of the stiffness modulus, and, thus, establish the low-temperature constitutive relationship. These models are directly related to the low-temperature mechanical behavior of modified asphalt. The performance improvements such as enhanced deformation resistance and creep resistance brought by modification are reflected through changes in model parameters (e.g., increased elastic modulus, improved viscosity coefficient, etc.), which provides a basis for evaluating low-temperature durability.

#### 3.2.1. Burgers Model

The Burgers model is a viscoelastic constitutive model that is widely used to describe the nonlinear elastic and viscous deformation behaviors of solid materials [35]. Its basic assumption is that the material is regarded as a series composite composed of elastic bodies and viscous bodies with different relaxation times. The model is formed by connecting elastic elements and viscous elements in series, and its expression is shown in Equation (1). The elastic element represents the elastic recovery of the material after being subjected to force, while the viscous element describes the viscous relaxation of the material after being stressed. In the Burgers model, the expression of the elastic element is similar to that in the standard elastic model, and it is usually simplified to the expression of the linear Hooke’s law, whereas the viscous element adopts the expression of the Maxwell linear viscoelastic model.

(1) $$\varepsilon \left(t\right)=\sigma \left[\frac{1}{E_{1}}+\frac{1}{\eta_{1}}t+\frac{1}{E_{2}}(1-e^{-\frac{E_{2}}{\eta_{2}}t})\right]$$

where $\varepsilon \left(t\right)$ is the strain at time *t*; σ is the applied stress; t is time; $E_{1}$ and $E_{2}$ are the elastic parameters of the model; $\eta_{1}$ and $\eta_{2}$ are the viscous parameters of the model.

Then, Equation (1) is simplified by dividing both sides of the equation by σ, which results in Equation (2).

(2) $$J\left(t\right)=\frac{1}{E_{1}}+\frac{1}{\eta_{1}}t+\frac{1}{E_{2}}(1-e^{-\frac{E_{2}}{\eta_{2}}t})$$

where $J\left(t\right)$ is the creep compliance at time of *t*, MPa^−1^.

**Figure 3:**
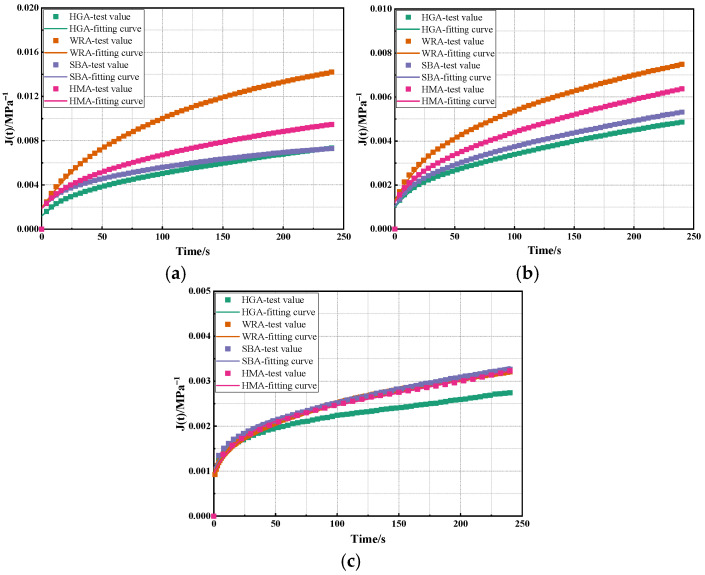
The calculation results of the Burgers model: (**a**) −6 °C; (**b**) −12 °C; (**c**) −18 °C.

Based on the Burgers model, calculations were conducted on the creep curves of several high-modulus asphalts obtained from BBR tests at different temperatures, with the results being shown in Figure 3 and Table 2.

As shown in Figure 3 and Table 2, the Burgers model exhibits high fitting accuracy for the BBR test results of several high-modulus asphalts. It can be found that the R^2^ of the fitted curves for these high-modulus asphalts at different temperatures are significantly higher than 0.9, which indicates that the variation trend of the low-temperature performance of high-modulus asphalt conforms to the nonlinear curve relationship established by the Burgers model. Furthermore, as the temperature decreases, E_1_, η_1_, E_2_, and η_2_ are all observed to gradually increase. This suggests that asphalt tends to exhibit more elastic behavior with decreasing temperature, mainly because lower temperatures cause asphalt to gradually become more viscous and brittle-hard, with its flow performance being significantly reduced. Consequently, it is more prone to brittle fracture at low temperatures, and the low-temperature performance of asphalt gradually deteriorates as the temperature decreases, which is consistent with the calculation results of low-temperature creep characteristic parameters mentioned earlier. Furthermore, it can be observed that, when the temperature is higher than −18 °C, the overall variation trend of the Burgers model parameters for several high-modulus asphalts is WRA < HMA < SBA < HGA. This indicates that waste rubber powder-modified asphalt has a lower impact on the viscoelastic properties of asphalt, and the proportion of viscoelastic components in it is relatively higher than that in other high-modulus asphalts. However, the asphalt with the lowest proportion of internal viscoelastic components is HGA. This is because HGA is a hard asphalt, and its enhanced high modulus stems from its high proportion of internal elastic components, which results in HGA having the poorest low-temperature performance. However, when the temperature reaches −18 °C, the overall variation trend of the Burgers model parameters for several high-modulus asphalts is SBA < WRA < HMA < HGA, which is related to the BBR test results of these high-modulus asphalts. It can be observed that, when the test temperature reaches −18 °C, the order of S values for the several high-modulus asphalts is SBA < WRA < HMA < HGA. Constitutive models essentially involve fitting calculations on the time–history curves of the creep compliance of asphalt at low temperatures, and the creep compliance is derived from the time–history curves of S values. This also means that the Burgers model effectively reflects the creep behavior characteristics of high-modulus asphalt at low temperatures.

#### 3.2.2. TPS Model

The TPS model is a viscoelastic constitutive model used to describe materials. Based on the Burgers model, this model introduces an additional dissipation coefficient to more accurately characterize the viscous properties of materials, with its expression given in Equation (3) [36]. The TPS model consists of one elastic element and two viscous elements connected in series. Among them, one viscous element is used to describe the rapid relaxation behavior on a short time scale, the other viscous element is employed to characterize the slow relaxation behavior on a long time scale, and the elastic element is responsible for describing the elastic recovery of the material.

(3) $$\varepsilon (t)=\sigma \left\{\frac{1}{E_{1}}+\frac{1}{E_{2}}\left[1-exp(-\frac{t}{\tau_{d}})\right]\right\}$$

Equation (3) is further processed by dividing both sides by σ, and the result is shown in Equation (4).

(4) $$J(t)=\frac{1}{E_{1}}+\frac{1}{E_{2}}\left[1-exp(-\frac{t}{\tau_{d}})\right]$$

where $\tau_{d}$ is the viscous parameter of the TPS model.

**Figure 4:**
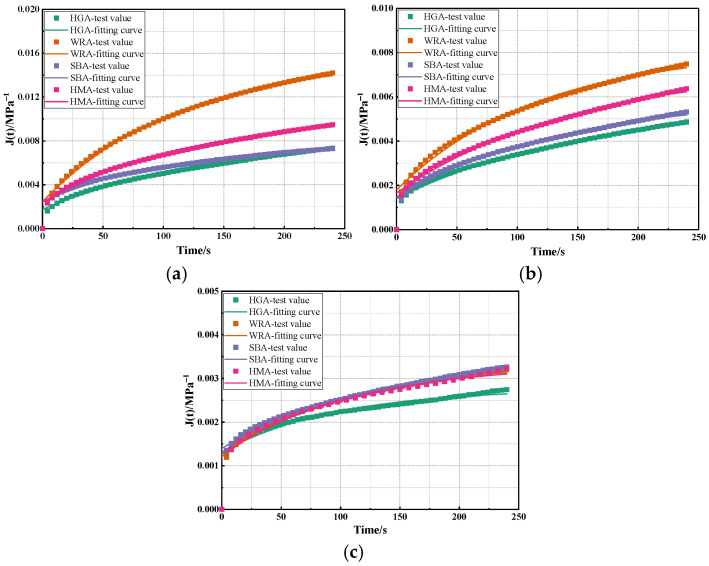
The calculation results of the TSP model: (**a**) −6 °C; (**b**) −12 °C; (**c**) −18 °C.

Based on the TPS model, calculations were conducted on the creep curves of several high-modulus asphalts obtained from BBR tests at different temperatures, with the results being shown in Figure 4 and Table 3.

As shown in Figure 4 and Table 3, the fitting accuracy of the curves from the TPS model for the BBR tests of several high-modulus asphalts are all higher than 0.9, which indicates that the TPS model can characterize the creep laws of high-modulus asphalt under stress at low temperatures to a certain extent. In addition, as the temperature decreases, it can be found that both E_1_ and E_2_ increase significantly, which is similar to the variation trend of the elastic parameters in the Burgers model. Interestingly, τ_d_ does not undergo obvious changes with decreasing temperature, which means that the TPS model cannot effectively characterize the influence of temperature on the viscous state of high-modulus asphalt. In addition, although the fitting accuracy of the TPS model is higher than 0.9, the R^2^ values of its fitted curves are significantly lower than those of the Burgers model, which further confirms that the TPS model is inferior to the Burgers model in evaluating the low-temperature performance of high-modulus asphalt. Furthermore, it can be observed that, when the temperature is higher than −18 °C, the overall variation trend of the elastic parameters of the TPS model for several high-modulus asphalts is WRA < HMA < SBA < HGA, which is consistent with the analysis results of the Burgers model. However, when the temperature reaches −18 °C, the variation trend of the parameters of the TPS model for several high-modulus asphalts is extremely disordered. Different from the Burgers model, it does not effectively reflect the variation law of the S values of high-modulus asphalt. This indicates that, as the temperature continues to decrease, the TPS model also struggles to effectively characterize the viscoelastic variation laws of high-modulus asphalt at low temperatures.

#### 3.2.3. FPS Model

The FPS model is a viscoelastic constitutive model used to describe materials. Compared with the TPS model, it introduces additional parameters to more accurately describe the mechanical behavior of materials [37]. The FPS model is composed of one elastic element and three viscous elements connected in series, with each element consisting of a spring and a damper to characterize the elastic and viscous properties of materials. The model’s expression is shown in Equation (5).

(5) $$\varepsilon \left(t\right)=\sigma \left\{\frac{1}{E_{1}}\left[1-exp(-\frac{E_{1}t}{\eta_{1}})\right]+\frac{1}{E_{2}}\left[1-exp(-\frac{E_{2}t}{\eta_{2}})\right]\right\}$$

Equation (3) is further processed, and by dividing both sides by σ, the result in Equation (6) is obtained.

(6) $$J(t)=\frac{1}{E_{1}}\left[1-exp(-\frac{E_{1}t}{\eta_{1}})\right]+\frac{1}{E_{2}}\left[1-exp(-\frac{E_{2}t}{\eta_{2}})\right]$$

**Figure 5:**
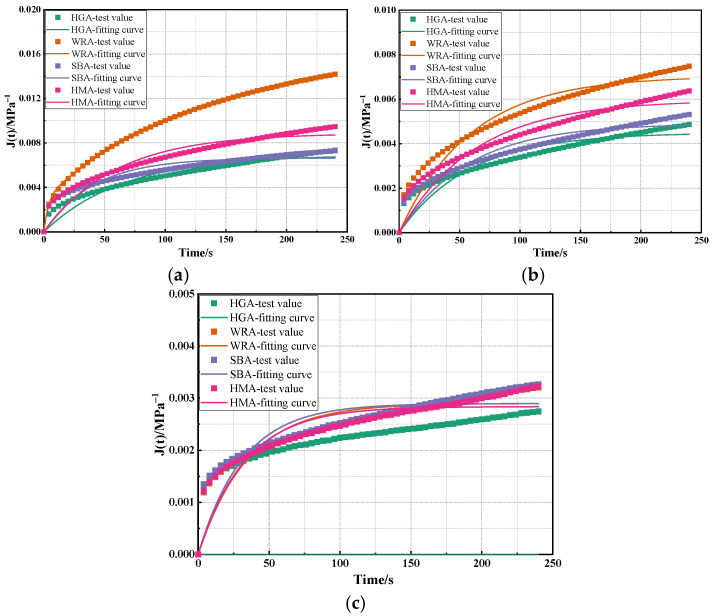
The calculation results of the FSP model: (**a**) −6 °C; (**b**) −12 °C; (**c**) −18 °C.

Based on the FPS model, calculations were conducted on the creep curves of several high-modulus asphalts obtained from BBR tests at different temperatures, with the results being shown in Figure 5 and Table 4.

As shown in Figure 5 and Table 4, the FPS model exhibits poor fitting accuracy for the BBR test results of several high-modulus asphalts. It can be found that the R^2^ values of most fitted curves are significantly lower than 0.9, which indicates that the FPS model is unable to effectively characterize the creep behavior of high-modulus asphalt at low temperatures. It is observed that the viscoelastic parameters of each asphalt’s constitutive model, calculated by the FPS model, show significant fluctuations and, when the temperature or asphalt type changes, the model parameters do not present an obvious variation pattern. In addition, the constitutive model parameters of some asphalts even take negative values, which means that the constraint conditions set by the FPS model are not applicable to the low-temperature performance test results of high-modulus asphalt. Finally, considering the fitting accuracy of the three constitutive models and the variation patterns of their parameters, it can be concluded that the Burgers model is more suitable for evaluating the temperature-dependent creep behavior of high-modulus asphalt at low temperatures.

#### 3.2.4. Prediction Result

**Figure 6:**
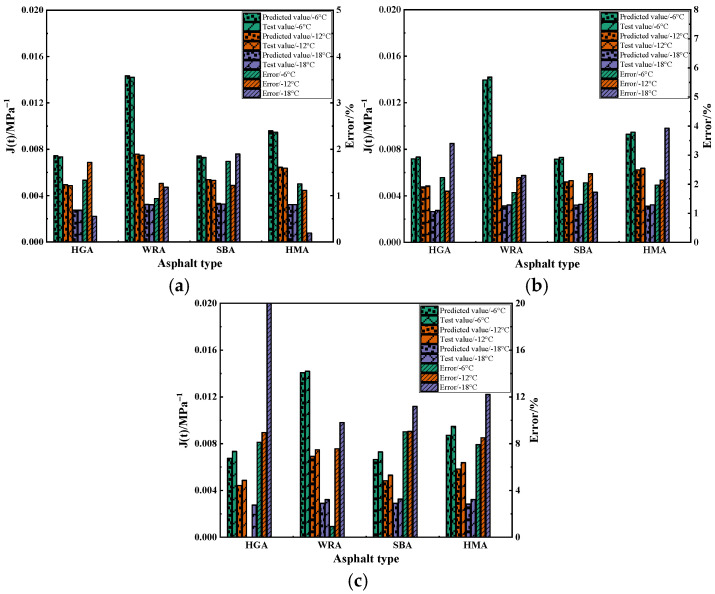
The calculation results of three models: (**a**) Burgers model; (**b**) TPS model; (**c**) FPS model.

This study respectively employed the Burgers model, TPS model, and FPS model to evaluate the low-temperature creep behavior of several high-modulus asphalts at different temperatures. It was found that, for the low-temperature performance of high-modulus asphalt, the Burgers model exhibits higher fitting accuracy, and the variation in its parameters is more regular. Therefore, compared with the other two constitutive models, the Burgers model is considered more suitable for evaluating the low-temperature performance of high-modulus asphalt. However, this analysis is only from the perspectives of fitting curve accuracy and parameter variation rules. The applicability of a constitutive model should be further analyzed through its prediction effect. For this purpose, this study took the final values of the creep stage from BBR tests of several high-modulus asphalts at different temperatures as the benchmark, evaluated the applicability of several constitutive models, and analyzed the error between the predicted values and test values of these models, with the results being shown in Figure 6.

As shown in Figure 6, as the temperature decreases, the error values of the several models all exhibit a certain degree of fluctuation. However, compared with the TPS model and FPS model, the error of the Burgers model is the least affected by temperature changes, which indicates that the Burgers model has the most stable prediction effect on the low-temperature performance of high-modulus asphalt. Furthermore, it can be found that changes in both asphalt type and temperature have the smallest impact on the Burgers model. The maximum error value of the Burgers model does not exceed 2%, while the error values of the TPS model and FPS model exceed 4% and 20%, respectively. This further demonstrates that the Burgers model is more capable of effectively describing the creep behavior of high-modulus asphalt at low temperatures than the other two models. Interestingly, similar to the variation law of low-temperature creep parameters, regardless of the constitutive model used, the error values of several high-modulus asphalts generally show that WRA is the lowest, followed by HMA or SBA, and that HGA is the highest. This indicates that the high-modulus asphalt prepared with waste rubber powder has more stable service performance at low temperatures, followed by high-modulus asphalt prepared with polymers and hard asphalt, which is consistent with the calculation results of low-temperature creep parameters.

The preceding analysis assessed the applicability of several constitutive models by examining the error characteristics between predicted values and test values. However, it was found that the error values of the constitutive models exhibit a certain degree of fluctuation with changes in asphalt type and temperature. Therefore, there remains a limitation in analyzing the applicability of constitutive models solely based on the overall variation trend. For this, this study employs the GRA model to further explore the constitutive models suitable for evaluating the low-temperature performance of high-modulus asphalt [38,39]. To simplify the analysis process, considering that the low-temperature limit temperature of several high-modulus asphalts was −12 °C in the calculation and analysis of low-temperature creep parameters, this study takes the error values of the three constitutive models at −12 °C as the reference sequence (denoted as X_01_~X_03_, wherein X_01_ is the error value of the Burgers model, X_02_ is that of the TPS model, and X_03_ is that of the FPS model). Then, the asphalt types are taken as the comparison sequence, with the calculation methods being shown in Equations (7)–(14).

(7) $$X_{0}=\left\{X_{0}\left(k\right)\left|k=1,2,\cdots n.\right.\right\}$$

(8) $$X_{i}=\left\{X_{i}\left(k\right)\left|k=1,2,\cdots n.\right.\right\}$$

(9) $$Y_{0}=\left\{Y_{0}\left(k\right)\right\}=\left\{X_{0}\left(k\right)/{\bar{X}}_{0}\left|k=1,2,\cdots n.\right.\right\}\;$$

(10) $$Y_{i}=\left\{Y_{i}\left(k\right)\right\}=\left\{X_{i}\left(k\right)/{\bar{X}}_{i}\left|k=1,2,\cdots n.\right.\right\}$$

(11) $$\bar{X_{i}}=\frac{\sum_{k=1}^{n}X_{0}\left(k\right)}{N}\left(i=1,2\ldots n\right)$$

(12) $$X_{i}=\frac{\sum_{k=1}^{n}X_{i}\left(k\right)}{N}(i=1,2\ldots n)$$

(13) $$\varepsilon_{i}\left(k\right)=\frac{\underset{i}{min}\underset{k}{min}\left|Y_{0}\left(k\right)-Y_{i}\left(k\right)\right|+\varepsilon *\underset{i}{max}\underset{k}{max}\left|Y_{0}\left(k\right)-Y_{i}\left(k\right)\right|}{\left|Y_{0}\left(k\right)-Y_{i}\left(k\right)\right|+\varepsilon *\underset{i}{max}\underset{k}{max}\left|Y_{0}\left(k\right)-Y_{i}\left(k\right)\right|}$$

(14) $$\gamma_{i}=\frac{1}{N}\sum_{k=1}^{n}\varepsilon_{i}(k)$$

where $X_{0}$ is the reference sequence; $X_{i}$ is the comparison sequence; $Y_{0}$ and $Y_{i}$ are the results of normalization processing for $X_{0}$ and $X_{i}$; $\varepsilon_{i}\left(k\right)$ is the correlation coefficient of $X_{0}$ and $X_{i}$ at time k; ε is the resolution coefficient; $\underset{i}{min}\underset{k}{min}\left|Y_{0}\left(k\right)-Y_{i}\left(k\right)\right|$ is the minimum difference of two levels; $\underset{i}{max}\underset{k}{max}\left|Y_{0}\left(k\right)-Y_{i}\left(k\right)\right|$ is the maximum difference of two levels; $\gamma_{i}$ is the correlation degree.

The present study takes asphalt types as the comparison sequences and the error values of the constitutive models as the reference sequences to evaluate the correlation between several constitutive models and high-modulus asphalt, with the results being presented in Table 5, Table 6, Table 7 and Table 8.

As shown in Table 5, Table 6, Table 7 and Table 8, the calculated correlation degree of the Burgers model is the highest, followed by the TPS model and the FPS model, which is consistent with the previous analysis results. This indicates that, compared with the TPS model and FPS model, the Burgers model is more suitable for evaluating the creep behavior of high-modulus asphalt at low temperatures, and thus effectively characterizes the low-temperature performance of high-modulus asphalt. Interestingly, there are some differences from the previous analysis results regarding error values. The GRA model suggests that WRA has the highest correlation degree with the Burgers model, followed by SBA, HMA, and HGA. This means that, according to the GRA model results, the Burgers model is more suitable for evaluating the low-temperature performance of WRA, followed by SBA and HMA, while its evaluation effect on hard asphalt is relatively poor. Overall, although there are certain differences between the GRA model results and the previous analysis results, both indicate that the Burgers model is more suitable for evaluating the low-temperature performance of high-modulus asphalt.

## 4. Conclusions

This study selected four representative high-modulus asphalts as research subjects, measured their creep characteristic parameters in low-temperature environments via the BBR test, and systematically explored the low-temperature viscoelastic constitutive behavior of high-modulus asphalt using three typical viscoelastic constitutive models. To screen out the model most suitable for characterizing the low-temperature creep properties of high-modulus asphalt, the GRA model was further adopted to conduct a quantitative evaluation of the three constitutive models using dimensions such as parameter stability, fitting accuracy, and significance of physical meaning. The main conclusions are as follows:

(1) A decrease in temperature leads to the deterioration of the low-temperature performance of high-modulus asphalt. The low-temperature limit temperatures of WRA and HMA are −12 °C, while those of SBA and HGA are −6 °C. However, compared with HGA, the value of SBA is extremely close to the specification value. There are certain limitations in evaluating low-temperature performance solely by the S value or m value; the S/m index can be used to better evaluate the low-temperature performance of high-modulus asphalt, and the variation law of its value is similar to the S value.
(2) Compared with the TPS model and FPS model, the Burgers model exhibits the highest fitting accuracy for the creep behavior of high-modulus asphalt at low temperatures, with the least fluctuation in its parameters. Moreover, the variation law of viscoelasticity of high-modulus asphalt at different temperatures characterized by the Burgers model is the most pronounced.
(3) The results of error analysis between the predicted values and measured values of several constitutive models also indicate that the prediction error of the Burgers model is significantly lower than those of the TPS model and FPS model. Additionally, all constitutive models show that the error of WRA is the lowest, followed by HMA, SBA, and HGA, which is consistent with the variation law of low-temperature creep parameters.
(4) The calculation results of the GRA model indicate that the Burgers model has a higher correlation degree with the creep behavior of high-modulus asphalt at low temperatures. Compared with the other two models, the Burgers model is more suitable for evaluating the low-temperature performance of high-modulus asphalt, and can provide a reference for the quantitative analysis of the low-temperature performance of high-modulus asphalt.

## Figures and Tables

**Table 1:** Basic properties of asphalt.

Properties	Index	HMA	SBA	WRA	HGA	Test Method
Penetration	(25 °C)/0.1 mm	28.5	33.6	27.2	24.2	JTG E20-2011 T 0604
Ductility	(10 °C)/cm	22.3	32.5	25.1	20.1	JTG E20-2011 T 0605
Softening point	°C	71.6	77.2	71.2	70.9	JTG E20-2011 T 0606
RTFOT (163 °C, 85 min)	Mass loss rate/%	−0.44	−0.41	−0.40	−0.45	JTG E20-2011 T 0610
	Penetration ratio/%	75	73	75	76	JTG E20-2011 T 0604
	Residual ductility/cm	11.6	14.7	10.7	8.4	JTG E20-2011 T 0605

**Table 2:** The parameter values of the Burgers model.

Model Parameters		HGA	WRA	SBA	HMA
−6 °C	E_1_	843.19615	523.64022	534.3155	536.70143
	η_1_	59,824.97044	40,339.74074	79,204.95315	52,146.81046
	E_2_	446.37031	153.36513	395.96868	319.9317
	η_2_	12,161.83696	7459.04435	10,643.31912	10,830.59752
	R^2^	0.99764	0.99833	0.99294	0.99671
−12 °C	E_1_	1010.12765	761.27996	962.75842	822.11992
	η_1_	91,303.20848	68,275.24392	87,269.4383	70,915.57502
	E_2_	754.7721	363.93267	630.48916	543.28204
	η_2_	17,547.85839	12,264.96283	16,139.76614	15,919.87678
	R^2^	0.99607	0.99708	0.99651	0.99704
−18 °C	E_1_	1025.736	1019.55527	951.38468	1047.96208
	η_1_	257,328.4355	198,436.2919	172,404.0699	184,948.0872
	E_2_	1179.80417	945.33739	1138.13827	1028.86806
	η_2_	18,732.5192	26,459.86258	19,650.05873	21,651.39941
	R^2^	0.98204	0.99006	0.98775	0.99037

**Table 3:** The parameter values of the TSP model.

Model Parameters		HGA	WRA	SBA	HMA
−6 °C	E_1_	567.06645	406.65725	407.97014	410.86885
	E_2_	148.77111	76.41055	191.30032	122.57974
	τ_d_	146.64497	113.83196	104.6702	131.13316
	R^2^	0.99075	0.99592	0.98386	0.99168
−12 °C	E_1_	712.50906	557.60436	678.54667	605.2104
	E_2_	231.82746	157.61321	220.07611	173.54951
	τ_d_	157.80491	117.59354	141.45287	151.4009
	R^2^	0.98899	0.99129	0.98871	0.99137
−18 °C	E_1_	764.07847	828.49909	716.73367	791.87438
	E_2_	686.81291	469.41071	465.0954	479.96828
	τ_d_	95.45648	101.69085	130.44237	111.72521
	R^2^	0.95521	0.98146	0.97505	0.97522

**Table 4:** The parameter values of the FSP model.

Model Parameters		HGA	WRA	SBA	HMA
−6 °C	E_1_	144.48861	331.1094	−3.90×10^30^	−3.50×10^21^
	η_1_	9461.13418	1203.21864	−3.86×10^30^	−2.82×10^23^
	E_2_	−1.33×10^27^	75.85964	150.13616	112.67177
	η_2_	9.66×10^28^	9992.47915	6119.37899	6673.80092
	R^2^	0.90944	0.99916	0.81706	0.89807
−12 °C	E_1_	222.38328	142.19823	203.99135	93.65979
	η_1_	12,885.31828	8344.29964	11,616.59857	5875.18806
	E_2_	−5.10×10^28^	8.04×10^21^	−9.50×10^28^	−211.92218
	η_2_	−4.13×10^31^	−2.96×10^23^	−1.45×10^32^	−13,299.3926
	R^2^	0.86093	0.91429	0.87453	0.89224
−18 °C	E_1_	−1.17×10^19^	−3.41×10^20^	98.18547	−5.08×10^30^
	η_1_	1.69×10^20^	−8.61×10^22^	3123.79625	−8.33×10^31^
	E_2_	−5.23×10^19^	345.0442	−137.21308	352.41685
	η_2_	−3.04×10^7^	12,027.63231	−4383.83914	11,617.15667
	R^2^	0.23742	0.75781	0.65102	0.71959

**Table 5:** Raw sequence of asphalt.

Variable	HGA	WRA	SBA	HMA
X_01_	1.71682735	1.260727086	1.218859306	1.111892276
X_02_	1.760608809	2.221770136	2.35845426	2.140113272
X_03_	8.944678975	7.557351148	9.044923215	8.500398057
X_1_	1	1	1	1

**Table 6:** The sequence after equalization.

Variable	HGA	WRA	SBA	HMA
X_01_	1.293691316	0.95000332	0.918454439	0.837850925
X_02_	0.830383172	1.047887823	1.112354271	1.009374733
X_03_	1.050851665	0.887863618	1.062628703	0.998656014
X_1_	1	1	1	1

**Table 7:** Absolute difference between two sequences.

Variable	HGA	WRA	SBA	HMA
△_011_	0.293691316	0.04999668	0.081545561	0.162149075
△_021_	0.169616828	0.047887823	0.112354271	0.009374733
△_031_	0.050851665	0.112136382	0.062628703	0.001343986

**Table 8:** Results of GRA.

Variable	HGA	WRA	SBA	HMA	γ
ε_011_	0.446823648	1	0.861864735	0.637041077	0.736432365
ε_021_	0.370180043	0.709765016	0.477692556	1	0.639409404
ε_031_	0.536964594	0.341323521	0.483687273	1	0.590493847

## Data Availability

The original contributions presented in this study are included in the article. Further inquiries can be directed to the corresponding authors.

## References

[1] Wang X., Geng L., Li K., Xu Q., Ding Y., Tao Y. (2024). Cracking Propagation of Asphalt Pavement of Stabilized Base with Inorganic Binder under Coupling of Overloaded Traffic and Temperature. J. Transp. Eng. Part B Pavements.

[2] Hu X., Sun L., Hu S., Walubita L.F. (2008). A simple and effective laboratory test device for measuring tire-pavement contact pressure. J. Test. Eval..

[3] Abiola O.S., Kupolati W.K., Sadiku E.R., Ndambuki J.M. (2014). Utilisation of natural fibre as modifier in bituminous mixes: A review. Constr. Build. Mater..

[4] Zhao F., Tang Y., Wu J., Huang Z., Gao M., Long Y. (2021). Mechanical Characteristics of Asphalt Pavement Pothole Maintenance. J. Eng..

[5] Kozel M., Remek Ľ., Ďurínová M., Šedivý Š., Šrámek J., Danišovič P., Hostačná V. (2021). Economic Impact Analysis of the Application of Different Pavement Performance Models on First-Class Roads with Selected Repair Technology. Appl. Sci..

[6] Bian F., Cai H. (2012). Choice of crack repairing material for asphalt pavement based on AHP. J. Test. Eval..

[7] Wang Y., Kong L., Chen Q., Lau B., Wang Y. (2017). Research and application of a black rapid repair concrete for municipal pavement rehabilitation around manholes. Constr. Build. Mater..

[8] Hand A.J., Ragavan P., Elias N.G., Hajj E.Y., Sebaaly P.E. (2022). Evaluation of Low Volume Roads Surfaced with 100% RAP Millings. Materials.

[9] Wang X., Qiu Y.J., Xue S.Y., Yang Y., Zheng Y. (2018). Study on durability of high-modulus asphalt mixture based on TLA and fibre composite modification technology. Int. J. Pavement Eng..

[10] Liu B., Xia C., Liu Y., Lv S., Yao H., Zhang N., Zhao S., Liu T. (2022). Uniform fatigue characterization of high modulus asphalt mixtures under three-dimensional stress state. Constr. Build. Mater..

[11] Ma T., Ding X., Zhang D., Huang X., Chen J. (2016). Experimental study of recycled asphalt concrete modified by high-modulus agent. Constr. Build. Mater..

[12] Pszczola M., Rys D., Jaczewski M. (2022). Field evaluation of high modulus asphalt concrete resistance to low-temperature cracking. Materials.

[13] Li Q., Shen A., Wang L., Guo Y., Wu J. (2024). Long-Term Performance of Modified Nature Asphalt–Derived High Modulus Asphalt Mixtures under Heavy Loads and Humid-Hot Climates. J. Mater. Civ. Eng..

[14] Hajikarimi P., Aflaki S., Hosseini A.S. (2013). Implementing fractional viscoelastic model to evaluate low temperature characteristics of crumb rubber and gilsonite modified asphalt binders. Constr. Build. Mater..

[15] Walubita L.F., Fuentes L., Tanvir H., Chunduri H.R., Dessouky S. (2021). Correlating the asphalt-binder BBR test data to the HMA (ML-OT) fracture properties. J. Mater. Civ. Eng..

[16] Liu X., Cao F., Xiao F., Amirkhanian S. (2018). BBR and DSR testing of aging properties of polymer and polyphosphoric acid–modified asphalt binders. J. Mater. Civ. Eng..

[17] Mohammed A.M., Abed A.H. (2023). Enhancing asphalt binder performance through nano-SiO2 and nano-CaCO3 additives: Rheological and physical insights. Case Stud. Constr. Mater..

[18] Yan K., Liu W., You L., Ou J., Zhang M. (2021). Evaluation of waste cooling oil and European Rock Asphalt modified asphalt with laboratory tests and economic cost comparison. J. Clean. Prod..

[19] Zhang C., Wang H., You Z., Gao J., Irfan M. (2019). Performance test on Styrene-Butadiene-Styrene (SBS) modified asphalt based on the different evaluation methods. Appl. Sci..

[20] Notani M.A., Moghadas Nejad F., Fini E.H., Hajikarimi P. (2019). Low-temperature performance of toner-modified asphalt binder. J. Transp. Eng. Part B Pavements.

[21] Liu L., Huang Y., Liu Z. (2020). Laboratory evaluation of asphalt binder modified with crumb rubber and basalt fiber. Adv. Civ. Eng..

[22] Xu J., Fan Z., Lin J., Yang X., Wang D., Oeser M. (2021). Predicting the low-temperature performance of asphalt binder based on rheological model. Constr. Build. Mater..

[23] Marasteanu M.O., Cannone Falchetto A. (2018). Review of experimental characterisation and modelling of asphalt binders at low temperature. Int. J. Pavement Eng..

[24] Fu Z., Song R., Qin W., Shi K., Ma F., Li J., Li C. (2025). Investigation on the low temperature rheological properties of polymer modified asphalt. J. Traffic Transp. Eng. (Engl. Ed.).

[25] Cheng Y., Yu D., Tan G., Zhu C. (2018). Low-temperature performance and damage constitutive model of eco-friendly basalt fiber–diatomite-modified asphalt mixture under freeze–thaw cycles. Materials.

[26] Li Y., He X., Sun H., Tan Y. (2021). Research on viscoelastic-plastic damage characteristics of cement asphalt composite binder. Constr. Build. Mater..

[27] Chen S., Jin E., Xu G., Zhuo S., Chen X. (2022). Factors influencing the low-temperature properties of styrene-butadiene-styrene modified asphalt based on orthogonal tests. Polymers.

[28] Zhou J., Chen X., Xu G., Fu Q. (2019). Evaluation of low temperature performance for SBS/CR compound modified asphalt binders based on fractional viscoelastic model. Constr. Build. Mater..

[29] Wu Y. (2017). Low-temperature rheological behavior of ultraviolet irradiation aged matrix asphalt and rubber asphalt binders. Constr. Build. Mater..

[30] Zheng W., Yang Y., Chen Y., Yu Y. (2022). Low temperature performance evaluation of asphalt binders and mastics based on relaxation characteristics. Mater. Struct..

[31] Tan Y., Fu Y.K., Ji L., Zhang L. (2016). Low-temperature evaluation index of rubber asphalt. J. Harbin Inst. Technol..

[32] Sun L., Zhu Y. (2013). A serial two-stage viscoelastic–viscoplastic constitutive model with thermodynamical consistency for characterizing time-dependent deformation behavior of asphalt concrete mixtures. Constr. Build. Mater..

[33] Liu Y., Fan Y., Zhou Y., Chen B., Hu T., Wang H., Yang J., Huang W. (2025). Analysis of low temperature performance and viscoelastic properties of SBS-modified epoxy recycled asphalt. Int. J. Pavement Eng..

[34] Song J., Ouyang J., Ou J., Zheng J., Xu S. (2025). A constitutive model based on parallel rheological framework for large deformation and rate dependent behavior for SBS modified asphalt mortar. Constr. Build. Mater..

[35] Cao S., Li P., Nan X., Zhang X., Guo S. (2024). Evaluation of hard petroleum asphalt’s low-temperature performance and index optimisation based on the grey correlation model. Case Stud. Constr. Mater..

[36] Ye Y., Yang X., Chen C. (2009). Experimental comparison of creep models for asphalt sand at different levels of stresses. J. Huazhong Univ. Sci. Technol. (Nat. Sci. Ed.).

[37] Zhang W., Bahadori A., Shen S., Wu S., Muhunthan B., Mohammad L. (2018). Comparison of laboratory and field asphalt aging for polymer-modified and warm-mix asphalt binders. J. Mater. Civ. Eng..

[38] Zheng C., Li R., Hu M., Zou L. (2019). Determination of low-temperature crack control parameter of binding asphalt materials based on gray correlation analysis. Constr. Build. Mater..

[39] Tan Y., Zhang L., Xu H. (2012). Evaluation of low-temperature performance of asphalt paving mixtures. Cold Reg. Sci. Technol..

